# The Comorbidity of Depression and Diabetes Is Involved in the Decidual Protein Induced by Progesterone 1 (Depp1) Dysfunction in the Medial Prefrontal Cortex

**DOI:** 10.3390/metabo15010034

**Published:** 2025-01-09

**Authors:** Chen Xu, Mengxing Liao, Shize Zhang, Yuang Chen, Xinyue Shulai, Guangji Wang, Jiye Aa

**Affiliations:** Jiangsu Provincial Key Laboratory of Drug Metabolism and Pharmacokinetics, State Key Laboratory of Natural Medicines, China Pharmaceutical University, Nanjing 210009, China

**Keywords:** depression, diabetes, glucose metabolism, DEPP1, mPFC, synaptic protein

## Abstract

Background: There is a high rate of depressive symptoms such as irritability, anhedonia, fatigue, and hypersomnia in patients with type 2 diabetes mellitus (T2DM). However, the causes and underlying mechanisms of the comorbidity of depression and diabetes remain unknown. Methods: For the first time, we identified Decidual protein induced by progesterone 1 (Depp1), also known as DEPP autophagy regulator 1, as a hub gene in both depression and T2DM models. Depp1 levels were increased in the mPFC but not in other brain regions, such as the hippocampus or nucleus accumbens, according to Western blot and PCR assays. Results: Glucose dysregulation and synaptic loss occur in both depression and T2DM. The typical hyperglycemia in T2DM was observed in two models of depression, namely, chronic social defeat stress (CSDS) and chronic restraint stress (CRS). Hyperglycemia, which occurred in T2DM, was observed, and metabolomics data clearly showed the perturbation of glucose levels and glucose metabolism in the medial prefrontal cortex (mPFC). Decreased protein levels of BDNF and PSD95 suggested significant synaptic loss in depressed and diabetic mice. Conclusion: These findings suggest that the comorbidity of depression and diabetes is involved in the dysfunction of Depp1 in the mPFC.

## 1. Introduction

Major depressive disorder (MDD), a common mental disorder, can lead to serious self-harm or suicide, resulting in heavy mental and economic burdens on patients and their families [1,2]. Depression has been proposed as a metabolic disease that coexists with metabolic syndromes such as diabetes mellitus [3]. Studies indicate that the prevalence of diabetes in individuals with depression can be as high as 60%, significantly higher than in the general population [4]. The global prevalence of diabetes has increased as the population ages, and urbanization is projected to increase by more than 50% between 2017 and 2045 [5]. Notably, the incidence of depression in individuals with diabetes has tripled worldwide [4]. The comorbidity of diabetes and depression is increasingly common.

In comparable human studies, depressed patients with inhibited glucose metabolism in several subregions of the cortex [6] exhibit elevated basal blood glucose levels [7,8], which may be critically associated with the development of depression. Remarkably, the central nervous system depends on peripheral glucose uptake to meet its need for energy. Specifically, different types of stress-induced hyperglycemia [9,10,11,12] in animals disrupt the memory function in the brain due to inadequate glucose uptake [13,14]. Diabetes mellitus is a metabolic disease characterized by chronic hyperglycemia due to defective insulin secretion and/or utilization caused by multiple etiological factors [15]. Strict glycemic and dietary control and prolonged treatment aggravate a patient’s psychological burden, which in turn leads to depression [16]. Nevertheless, the mechanisms by which chronic stress affects glucose metabolism and how that disturbed glucose metabolism affects behavior in depression are unclear.

DEPP autophagy regulator 1 (Depp1), which is highly expressed in diverse tissues including the ovary, kidney, white adipose, and liver [17,18,19], is strongly induced by progesterone, androgens, and hypoxia [20,21]. The Forkhead Box O_3_/Depp1 pathway in neuroblastoma cells initially demonstrated its function in autophagy in mammals [22]. Multiple stresses and inflammation resulted in depressive-like behavior and dysregulated autophagy, which is essential for maintaining homeostasis and the function of the CNS [23]. BDNF (brain-derived neurotrophic factor), which played an important protective role in neurogenesis and synaptic plasticity, was significantly deleted by hyperactive neuronal autophagy in depressed mice [24]. In addition, overexpression of Depp1, which is negatively regulated by insulin, leads to ROS accumulation and plays a key role in the regulation of hepatic glucose and fat metabolism [25]. Depp1 loss is protective in the setting of chronic HIF activation and cardiac dysfunction [26]. The physiological functions of DEPP1 have not been fully delineated, especially in the nervous system and glucose metabolism disorder.

BDNF (brain-derived neurotrophic factor), postsynaptic density protein 95 (PSD-95), which played an important protective role in neurogenesis and synaptic plasticity, was significantly deleted in depressed mice [24,27]. In addition to disordered glucose metabolism, diabetes mellitus may lead to neuropathy [28] and inhibition of synaptic plasticity [29,30]. The expression of PSD-95, which is located in the postsynaptic density in excitatory synapses, was decreased by high glucose [31]. Therefore, in this study, we investigated the impact of Depp1 on the synthesis of synaptic proteins in both depression and diabetes models.

## 2. Materials and Methods

### 2.1. Animals

Male C57BL/6JNifdc mice (aged 6–7 weeks) and male CD1 aggressive mice (aged 6–8 months) were gathered from Vital River Laboratories in Beijing, China. All animals were housed under standard laboratory conditions with a 12 h light/dark cycle (lights were on from 7:00 to 19: 00), temperature 25 ± 1 °C, and the food and water ad libitum. All animal welfare and experimental protocols were strictly approved by the China Pharmaceutical University Animal Care and Use Committee (protocol code 2020-09-021).

### 2.2. Animal Models

#### 2.2.1. Chronic Social Defeat Stress

C57BL/6J mice were subjected to physical attacks from aggressive CD-1 mice for 10 min per day, followed by a reduction of 0.5 min per day for 10 days [32]. Following each 10 min attack session, the mice were segregated and housed on the opposite side of the cage to allow sensory contact for the next 24 h. The social interaction (SI) test was administered 24 h after the final attack.

#### 2.2.2. Chronic Restraint Stress

C57BL/6J mice were placed in a well-ventilated 50 mL polypropylene conical tube for 6 h (9:00–15:00) daily over a span of 28 consecutive days. Post-restraint, the mice were returned to their home cages with unrestricted access to food and water [33,34].

#### 2.2.3. Chronic Unpredicted Mild Stress

Mice were exposed to 2–3 stressors in random order per day, including cage tilting (45 °C 24 h), food deprivation (24 h), water deprivation (24 h), chronic social defeat stress, damp sawdust (12 h), restraint (6–12 h), tail clipping (10 min), forced swimming in 4 °C water, forced swimming in 40 °C water, and no bedding in the cage (24 h) [35].

#### 2.2.4. Induction of Type 2 Diabetes Mellitus

After 4 weeks of high-fat diet feeding (HFD, 60% energy from fat, 20% energy from protein, Cat. D12492, Jiangsu Xietong Pharmaceutical Bio-engineering Co., Ltd., Nanjing, China), mice were fasted for 12 h, followed by a single intraperitoneal injection of streptozocin (40 mg/kg) for 3 consecutive days. Then, the fasting blood glucose of mice was monitored every week. A blood glucose of more than 16.7 mmol/L is considered a successful diabetic model. Then, the fasting blood glucose of mice was monitored every week [36,37].

### 2.3. Behavioral Testing

#### 2.3.1. Social Interaction Test

Initially, the movement of mice was recorded for 2.5 min to estimate the time spent in the interaction zone without CD-1. A CD-1 mouse was then placed in the metal animal cage, and the time spent by the mice in the interaction zone with the CD-1 mouse was recorded. The social interaction (SI) ratio was calculated as the time spent in the interaction zone with the CD-1 mouse versus without the CD-1 mouse [38,39].

#### 2.3.2. Forced Swim Test

C57BL/6J mice were placed in a glass cylinder (27 cm in height, 18 cm in diameter) filled with 16 cm of water (23–26 °C) for 6 min. The immobility time of the mouse was calculated for the last 4 min [38,39].

#### 2.3.3. Tail Suspend Test

The tails of the mice were secured with tape in a tail suspension box, suspending them 25 cm above the ground for 6 min. The immobility time of the mouse was calculated for the last 4 min [38,39].

#### 2.3.4. Open Field Test

The locomotion and transfer of mice in an open field (50 height × 50 width × 35 cm depth) were recorded for 8 min. The distance and the time of mice between the central and periphery zones were recorded and analyzed by video tracking software (Any-maze, Video Tracking System 7.40) [38,39].

#### 2.3.5. Sucrose Preference Test

Mice were given a bottle of 2% sucrose solution and a bottle of pure water after water deprivation for 12 h. The sucrose and water intakes were measured after 12 h. Sucrose preference rate (%) = sucrose solution consumption/total liquid consumption × 100% [35].

#### 2.3.6. Coat Score Assay

The coat score was obtained by summing the scores for seven different body regions: head, neck, dorsal and ventral coat, tail, forepaws, and hindpaws. One point was awarded for a well-groomed coat in each region; otherwise, no points were given [39].

### 2.4. Blood Glucose Measurements

The mice were starved for 12 h, and the fasting blood glucose was measured in the morning with an electronic handheld glucometer (Accu-Chek; Roche, Indianapolis, IN, USA). Peripheral blood was obtained by tail-cut as described in previous studies [40].

### 2.5. RNA Isolation, qPCR, and RNA Sequencing

The isolation and extraction of total RNA were performed by Trizol (Takara, Kusatsu, Japan). cDNA was acquired from total RNA using the PrimeScript RT reagent kit (Takara). Quantitative real-time PCR was carried out using the SYBR Green mixture on a CFX96 Real-Time PCR cycler (Bio-Rad, Hercules, CA, USA). The primers are as follows: DEPP1 (Forward: 5′-CCCCATTTGCCAACGATTCG-3′; Reverse: 5′-GCTGACAGATACACCTGACGTAG-3′) and β-actin (Forward: 5′-ATGGAGGGGAATACAGCCC-3′; Reverse: 5′-TTCTTTGCAGCTCCTTCGTT-3′). The relative expression levels of DEPP1 mRNA were normalized to that of β-actin and determined by the ^△△^CT method [41].

After the behavioral assay, the bilateral medial prefrontal cortex tissue of the 3 control mice and 3 CSDS mice was immediately gathered for mRNA extraction. Purified cDNA fragments were sequenced using Illumina HiSeq TM 2500 by Gene Denovo Biotechnology Co, Guangzhou, China [39].

### 2.6. Western Blotting

To determine the expression of the protein, the hippocampus, hypothalamus, nucleus accumbens, medial prefrontal cortex, and dorsal raphe nucleus tissues were homogenized by ice-cold RIPA buffer (Beyotime, P0013B, Shanghai, China) containing 1% protease inhibitor and 1% phosphatase inhibitor (Beyotime). The sample was run, and protein bands were separated by SDS-PAGE. The gels, according to the different protein sizes, were transferred onto a PVDF membrane, and the blots were subsequently blocked for 1 h with 5% nonfat dry milk or BSA in TBS containing 0.1% Tween 20 at room temperature. After being transferred onto a PVDF membrane and incubated with primary antibodies to DEPP1 (1:1000, Proteintech, 25833-1-AP, Wuhan, China), β-tubulin (1:5000, Bioworlde, BS1482M, Dublin, OH, USA), PSD95 (1:1000, Abcam, ab2723, Waltham, MA, USA), and synaptophysin (1:50,000, Abcam, ab32127) overnight at 4 °C, the membranes were incubated with horseradish peroxidase-conjugated secondary antibodies (1:10,000) for 1.5 h at room temperature. The antibody-reactive bands were visualized by using enhanced chemiluminescence detection reagents (1:1) and a gel imaging system [38].

### 2.7. LC-Q/TOF-MS-Based Metabolomics Assays

According to our previous study, the hippocampus and mPFC of mice were extracted, weighed, and then extracted by 800 μL of 80% methanol (with 10 μg/mL internal standard, 4-Chloro-DL-phenylalanine), respectively. After homogenization and centrifugation at 18,000 rpm for 15 min, the supernatant (200 μL) was transferred and evaporated to complete dryness. ddH_2_O (100 μL) was added to the dried residue, and 10 μL supernatant was subjected to analysis using an HPLC Triple-TOF/MS 5600 system (AB SCIEX, Framingham, MA, USA). To identify the metabolites, the acquired accurate precursor (mass tolerance < 10 ppm), retention period, and fragment ions (mass tolerance < 20 ppm) should match with the standard metabolome databases, such as the Human Metabolome Database, MassBank, and METLIN metabolite database. Metabolite abundance was acquired by computing the area under the curve for the quantification ion of the metabolites with MultiQuant 3.0. The quantitative data were normalized by the internal standard and tissue weights simultaneously. The statistical analysis was performed using SIMCA-P 13 software (Umetrics, Umeå, Sweden) based on variable influence on projection (VIP) values [39]. All the raw data and analysis have been supplied in Tables S1 and S2.

### 2.8. Statistical Analyses

All results are presented as the mean ± SEM (standard error of the mean). Appropriate statistical tests were chosen depending on the normality of the data. An unpaired Student’s *t*-test was utilized to analyze the differences between the 2 groups by GraphPad Prism 9.0 software when the data followed the normal distribution. On the other hand, the two-tailed unpaired Mann–Whitney test (nonparametric test) was used. In all cases, a *p*-value lower than 0.05 was considered statistically significant [42]. All *p*-values are adjusted for multiple testing using FDR in metabolomics analysis (Table S2) and transcriptomics analysis (Table S4). Correlations were gauged with the Pearson correlation coefficient. All behavioral assays and analyses were performed by colleagues blinded to the experimental groups. All the specific information of statistical analysis has been shown in Table S3.

## 3. Results

### 3.1. Chronic Stress Leads to a Significant Elevation in Peripheral Glucose Levels and Depression-like Behaviors in Mice

**Figure 1:**
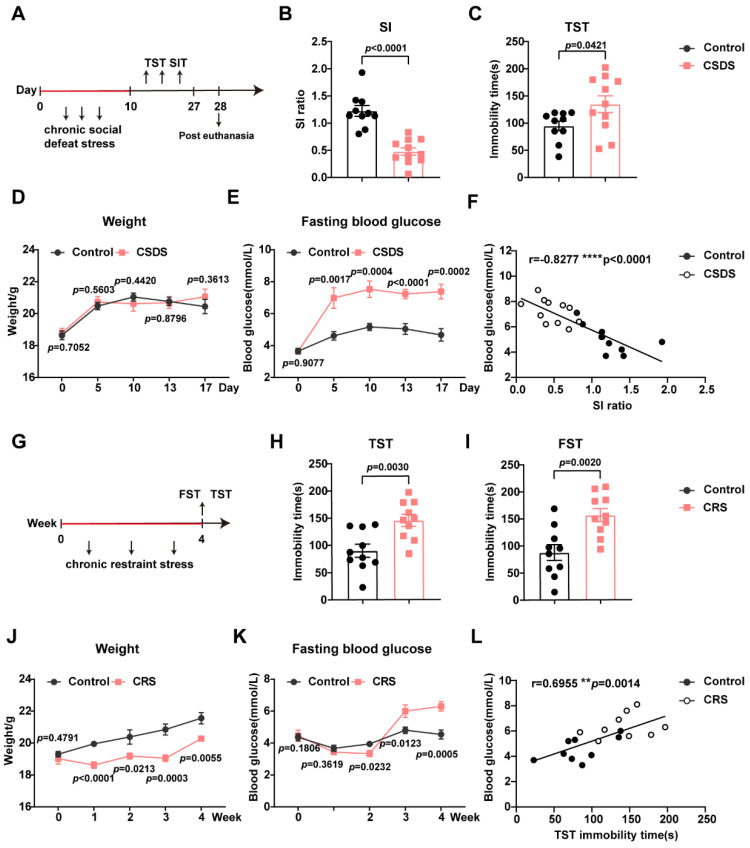
Hyperglycemia and depression-like behavior induced by chronic stress (**A**) A diagrammatic overview of chronic social defeat stress and behavioral assays. (**B**,**C**) CSDS facilitated depression-like behaviors, n = 10–11 in each group. (**D**) The weights of the animals, n = 10–11. (**E**) Peripheral hyperglycemia developed after 5 days of stress and lasted 1 week, n = 10–11 in each group. (**F**) Correlation of the value of fasting blood glucose value with SI ratio following CSDS, n = 11 or 10. (**G**) A diagrammatic overview of chronic restraint stress and behavioral assays. (**H**,**I**) CRS facilitated depression-like behaviors, n = 10 in each group. (**J**) The weights of the animals, n = 8–10. (**K**) Peripheral hyperglycemia developed after 3 weeks of stress and lasted 1 week, n = 9–10 in each group. (**L**) Correlation of fasting blood glucose with TST immobility time ratio following CSDS, n = 9. Data represent the mean ± SEM by two-tailed unpaired Student’s *t*-test. Correlations were gauged with the Pearson correlation coefficient, ** *p* < 0.01, **** *p* < 0.0001. CSDS, chronic social defeat stress; CRS, chronic restraint stress; SI, social interaction; TST, tail suspend test; FST, forced swim test.

Animal models suffering from different stresses can simulate stresses that humans experience in society. To comprehensively elucidate the profound effects of stress on energy metabolism, we constructed typical depression mice models and monitored fasting blood glucose levels throughout the entire modeling process. Chronic social defeat stress (CSDS) is widely accepted as a valid model of depression because of its reliability and reproducibility. In conformity with previous reports, in behavioral assays, we detected extended immobility in the tail suspension test (TST) and a diminished ratio in the social interaction test (SIT) for CSDS-exposed mice (Figure 1A–C), suggesting that CSDS induced significant depression-like behaviors. Fasting blood glucose (FBG) was significantly increased in model mice exposed to CSDS beginning on the fifth day of stress (Figure 1E), and this increase lasted until one week after the depression paradigm ended; moreover, no differences in weight (Figure 1D) were observed in the control mice and CSDS animals. Notably, correlation analysis in CSDS mice revealed that FBG levels were negatively correlated with social avoidance. Similarly, the chronic restraint stress model (CRS), which employs a non-invasive stimulus, simulates the development of depression in humans. After four weeks of restraint, there was a significant increase in immobility time in the FST and TST (Figure 1G–I), and the mice exhibited prominent despair behavior. CRS not only caused hyperglycemia (Figure 1K) but also decreased the weight (Figure 1J) of the mice during the last two weeks of restraint. Correlation analysis of CRS mice uncovered that FBG levels were positively correlated with immobility time in TST (Figure 1L). These results revealed that hyperglycemia was induced significantly in two different stress models.

### 3.2. There Was No Lasting Difference Despite a Temporary Increase in Peripheral Glucose Levels Between CUMS and Normal Mice

**Figure 2:**
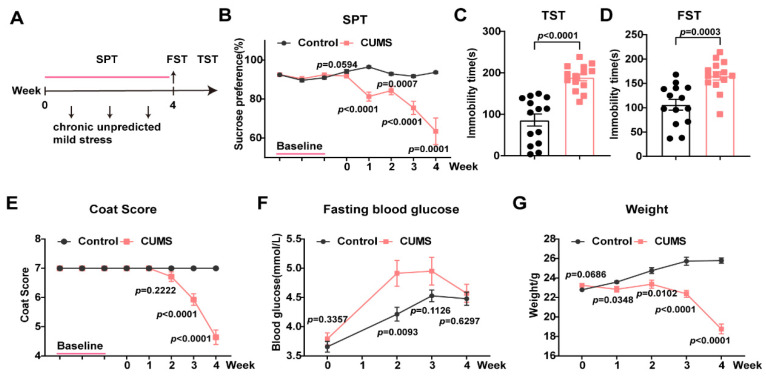
Fasting blood glucose levels caused by chronic unexpected mild stress. (**A**) A diagrammatic overview of chronic unexpected mild stress and behavioral assays. (**B**–**E**) CUMS facilitated depression-like behaviors, n = 13–14 in each group. (**F**) There was no difference in peripheral glucose levels between CUMS and normal mice despite an initial increase. n = 14 in each group. (**G**) The weights of animals. Data represent the mean ± SEM, by two-tailed unpaired Student’s *t*-test for (**B**–**G**), and by the two-tailed unpaired Mann–Whitney test (nonparametric test) for (**E**). CUMS, chronic unpredictable mild stress; SPT, sucrose preference test.

Multiple types of stressors are used over a long term in chronic unpredicted mild stress (CUMS) to better mimic the stresses that humans experience in society. Compared with nonstressed animals, mice subjected to CUMS exhibited lower sucrose preference (Figure 2A,B) in the sucrose preference test (SPT), longer immobility times in both the TST and FST (Figure 2C,D), and lower coat scores (Figure 2E), which are considered to be anhedonic and despair behaviors. During 4 consecutive weeks of CUMS, glucose levels initially tended to rise in stressed mice (week 2) but decreased to a level comparable to that of normal mice by the end of stress (Figure 2F). However, the weight of the mice was significantly reduced following the CUMS, probably due to prolonged exposure to different types of stress. Despite a temporary increase in peripheral glucose levels, the mice did not exhibit persistent hyperglycemia, as the CSDS and CRS groups did.

### 3.3. A High-Fat Diet and Streptozocin Induced Remarkable Depressive-like Behaviors in Mice

**Figure 3:**
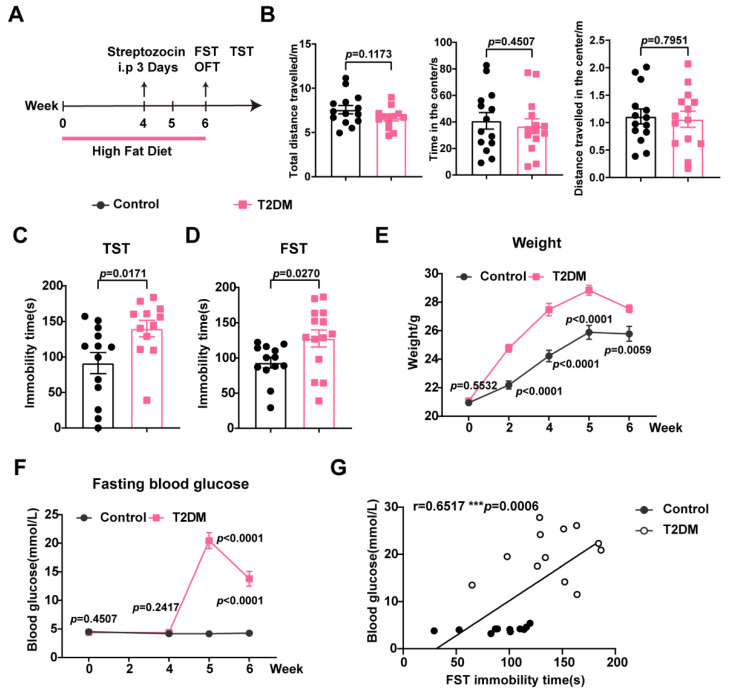
High-fat diet and streptozocin induced significant depressive-like behaviors and hyperglycemia in mice. (**A**) A diagrammatic overview of high-fat diet and behavioral assays. (**B**) No significant differences in locomotor activity, n = 6 in each group. (**C**,**D**) High-fat diet facilitated depression-like behaviors, n = 12–14 in each group. (**E**) The weight of animals, n = 14. (**F**) Peripheral hyperglycemia develops after being exposed to a high-fat diet and STZ, n = 13–14 in each group. (**G**) Correlation of the level of fasting blood glucose with the FST immobility time following T2DM, n = 12. Data represent the mean ± SEM by two-tailed unpaired Student’s *t*-test. Correlations were gauged with the Pearson correlation coefficient, *** *p* < 0.001. OFT, open field test; STZ, streptozocin; HFD, high-fat diet; T2DM, type 2 diabetes mellitus.

The above results regarding the elevated blood glucose observed in the depression model triggered great interest, i.e., whether the emotional behavior of diabetic patients suffering from hyperglycemia is influenced by levels of blood glucose. To evaluate the effects of high peripheral glucose levels on the behavior of animals, a high-fat diet (HFD) accompanied by a low dose of streptozocin (STZ) was used to establish a mouse model of T2DM. The open field test (OFT) was performed to describe a simple analysis of general locomotor ability. There were no significant differences in total distance, time in the center, and distance in the center between the two groups, indicating that HFD and STZ administration did not affect the locomotor activity of mice (Figure 3A,B). Whereas the HFD and STZ, in the T2DM model mice, significantly led to depressive-like behaviors (compared with normal controls), as evidenced by an increase in immobility time in the TST and FST (Figure 3C,D). Consistent with the previous studies, striking increases in weight (Figure 3E) and FBG levels (Figure 3F) were detected in T2DM mice. Correlation analysis of T2DM mice suggested that FBG levels were positively correlated with immobility time in the FST (Figure 3G).

### 3.4. Disorders of Glucose Metabolism in the Medial Prefrontal Cortex

**Figure 4:**
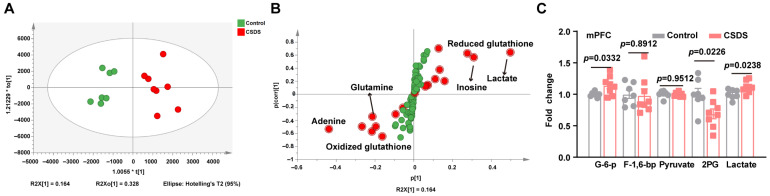
Metabolic patterns in the mPFC of CSDS mice. (**A**,**B**) OPLS-DA model of metabolic patterns in the mPFC. (**C**) The fold change in the effects of CSDS on the metabolites of glycolysis in the mPFC normalized to those in control mice, n = 7–9. Data represent the mean ± SEM by two-tailed unpaired Student’s *t*-test. mPFC, medial prefrontal cortex; G-6-P, glucose 6-phosphate; F-1,6-bp, Fructose 1,6-bisphosphate; 2-PG, 2-Phosphoglyceric acid; * *p* < 0.05.

Disordered peripheral glucose levels were observed in both depression and diabetes. Neurons, the most fundamental component of the nervous system, rely heavily on ATP generated by glucose metabolism for energy [43]. Disorders in glucose metabolism significantly influence the course of depression, as evidenced by diabetic patients having a more than 2-fold greater risk of developing depression than nondiabetic patients do. On the basis of untargeted metabolomics with HPLC-Q-TOF/MS, we screened the different metabolic profiles of the mPFC that contribute to depression and energy consumption. Orthogonal partial least squares discriminant analysis (OPLS-DA) loading plots of the mPFC (Figure 4A,B) showed different metabolic patterns between stressed animals and healthy mice. In detail, the elevated levels of glucose-6-phosphate and lactic acid, along with 2-glucuronic acid of the mPFC (Figure 4C), suggested impaired glucose metabolism in depressed mice, compared with that in control mice.

### 3.5. RNA Sequencing Identified Increased Expression of Depp1 in the Medial Prefrontal Cortex

**Figure 5:**
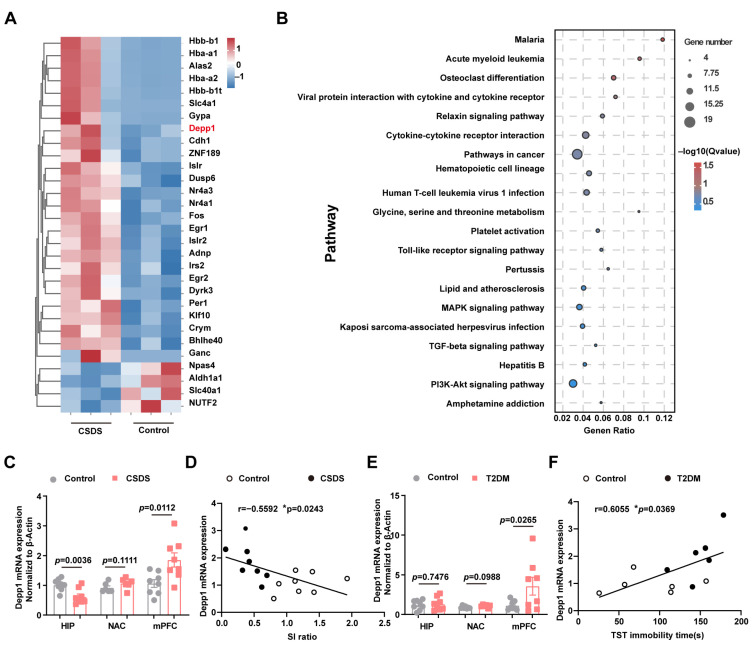
RNA sequencing was utilized to identify Depp1 in the medial prefrontal cortex as a hub gene in CSDS mice. (**A**) Heatmap representation of the 30 DEGs (FDR < 0.05) in control mice compared with CSDS mice, n = 3 in each group. (**B**) KEGG annotation of the differentially expressed genes (FDR < 0.05). (**C**) Depp1 mRNA levels in the brain region of CSDS mice compared with control mice, n = 6–8. (**D**) Correlation of Depp1 mRNA expression with the SI ratio following CSDS, n = 8. (**E**) Depp1 mRNA levels in the brain regions of T2DM mice and healthy mice, n = 6–8. (**F**) Correlation of Depp1 mRNA expression with TST immobility time following a high-fat diet and streptozocin, n = 6. Data represent the mean ± SEM, by two-tailed unpaired Student’s *t*-test for (**C**,**E**). Correlations were gauged with the Pearson correlation coefficient, * *p* < 0.05. HIP, hippocampus; NAC, nucleus accumbens.

Current investigations suggest that the interaction between depression and diabetes is bidirectional; however, further research is needed to identify key factors involved in the comorbidity of depression and diabetes and to make appropriate recommendations for treatment. Here, RNA sequencing was utilized to screen differentially expressed genes in the medial prefrontal cortex (mPFC) of depressive model animals (compared with nonstressed animals). A total of 41 differentially expressed genes (DEGs) were identified, of which 33 were upregulated and 8 were downregulated (FDR (adjusted *p*-value) < 0.05, fold change > 1.4) (Figure 5A, Table S4), and a variety of signaling pathways were strongly influenced (Figure 5B) in depressed mice. Depp1 (DEPP autophagy regulator 1) was highly expressed in CSDS mice, as shown by a PCR assay (Figure 5C).

Correlation analysis revealed that Depp1 mRNA levels were negatively correlated with social avoidance in CSDS mice (Figure 5D). Analogously, PCR analysis revealed significant overexpression in the mPFC of T2DM mice, which was positively correlated with TST immobility time (Figure 5E,F). These findings suggested that the comorbidity of depression and diabetes was involved in the dysfunction of Depp1 in the mPFC.

### 3.6. The Loss of Synaptic Proteins Was Observed in Both Depressed and Diabetic Mice

**Figure 6:**
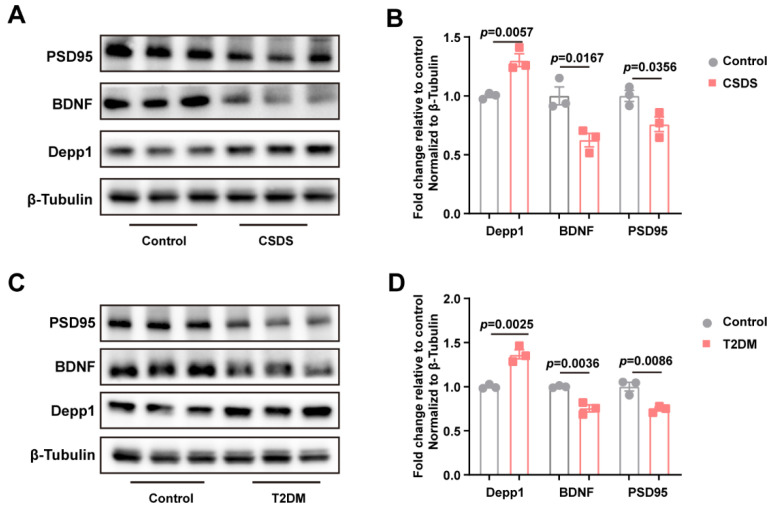
Upregulation of Depp1 along with loss of synaptic protein synthesis in both depressed and diabetic mice (**A**,**B**) The protein levels of Depp1, BDNF, and PSD95 in CSDS animals, n = 3. (**C**,**D**) The protein levels of Depp1, BDNF, and PSD95 in T2DM animals, n = 3. Data represent the mean ± SEM, by two-tailed unpaired Student’s *t*-test. BDNF, brain-derived neurotrophic factor; PSD95, postsynaptic density protein-95.

Besides the disorder of the central and peripheral glucose metabolism, what accounts for the depressive-like behavior in the brain in both depression and diabetes remains unknown. The expression of brain-derived neurotrophic factor (BDNF), synaptophysin (SYN), and postsynaptic density 95 (PSD95) is closely related to the regulation of neuroplasticity, especially synaptic changes in depression. Consistent with the data above, the protein content of Depp1 was markedly increased by chronic stress, with a significant decline in the levels of synaptic proteins, including BDNF and PSD95 (Figure 6A,B), which contributed to synaptic transmission. To our surprise, despite an increase in Depp1 in the mPFC, the protein levels of BDNF and PSD95 were reduced by hyperglycemia (Figure 6C,D).

## 4. Discussion

The comorbidity of depression and diabetes leads to increased rates of disability and death, as well as decreased quality of life, which is among the leading causes of health-related burdens [4]. There is an urgent need to elucidate the underlying mechanism involved in the co-occurrence of depression and diabetes.

In the present study, we compared peripheral blood glucose levels in mice exposed to different stresses that ultimately cause depression, including chronic social defeat stress (CSDS), chronic restraint stress (CRS), and chronic unexpected mild stress (CUMS). Different from CUMS mice, prolonged significant hyperglycemia was observed in CSDS and CRS mice. In agreement with the previous research on depressed animals, hyperglycemia was perceived in the early stage of CUMS [10,12]. Mice exposed to CUMS suffered from 2 to 3 different mild stresses daily, which caused the instability of modeling. In other words, peripheral glucose level alters and is associated with the duration and intensity level of unexpected stress.

Metabolomics, a depiction of overall metabolic patterns [44], has been widely applied to drug metabolism, pathology, and biomarker discovery by systematically identifying and quantifying all the metabolites in biological samples [45]. With the LC-Q/TOF-MS metabolomics assay, disturbed central glucose metabolism, as evidenced by the quantitation of metabolites in glycolysis, was detected in the hippocampus and medial prefrontal cortex of depressed mice. A decline in glucose metabolism has been reported in both depressed animals [46] and patients [47], especially in the mPFC, in which astrocytes contribute to the modulation of ATP and lactate [48]. The prevalence of depression in patients with diabetes is approximately 50% [3]. Therefore, a diabetic mouse model with STZ was constructed to examine its emotional behavior. Significant despair behavior, as shown by the TST and FST, was evident in the depression-like behavior of the T2DM mice induced by STZ. However, how disorders of peripheral and central glucose metabolism lead to severe depressive behaviors is still unknown.

To identify the pivotal targets of depression accompanied by hyperglycemia, RNA sequencing was utilized to screen differentially expressed genes in depressive models (compared with normal controls). Depp1, a DEPP autophagy regulator, is highly expressed in the mPFC of depressed mice and diabetic mice and potentially contributes to both despair behavior and hyperglycemia. Current studies on Depp1 have focused mainly on oxidative stress and autophagy in cancer [22] and ischemic cardiomyopathy [26]. Although overexpression of Depp1 in the liver reduces serum glucose and liver triglyceride (TG) levels [25], whether and how Depp1 acts in the brain remains unknown.

Disorders of central glucose metabolism in the hippocampus markedly lead to dysfunctions in synaptic transmission and cognition [49], further accelerating the progression of Alzheimer’s disease. Astrocytes deliver energy to the brain and provide substrates for many biological processes, thus playing a key role in maintaining glucose metabolism homeostasis [48]. Since astrocyte-derived ATP and lactate in the mPFC modulate depressive-like behaviors [43,50], glucose metabolism may play a more important role in synaptic transmission in the mPFC than in the hippocampus, which shows a more significant effect on learning and memory. mPFC, which serves as a critical upstream for circuits in depressive behavior, such as the mPFC-DRN pathway and mPFC-LHb pathways, integrates and transmits signaling projected to the cortex [50]. Other brain regions, such as the ventral tegmental area (VTA) and NAC, which mostly consist of dopamine neurons, play key regulatory roles in the stress response [51]. In accordance with the results of the synaptic protein levels in the mPFC of depressed animals, we previously reported that the protein levels of BDNF and PSD95 were reduced, which indicated a significant decline in synaptic transmission in the mPFC of diabetic animals. These findings suggest that Depp1 in the mPFC is a potential target for treating depression accompanied by hyperglycemia.

According to the previous results, the mechanisms underlying the comorbidity of depression and diabetes include insulin resistance, stress and hypothalamic–pituitary–adrenal axis, the neurological system, oxidative stress, and inflammation. Clinical first-line antidepressants, such as serotonin and norepinephrine reuptake inhibitors (SNRIs), partially improved despair behavior and glucose metabolism in patients with T2DM but risk complications [52]. Even if typical hypoglycemic agents, including metformin and pioglitazone, have the potential to reverse dysfunctions of glucose-metabolic- and depression-like behavior in depressed animals, they have not worked as well in clinical trials [52]. Above all, the search for natural drugs or small molecule compounds targeting DEPP1 may be a more comprehensive therapeutic approach for the treatment of comorbidities.

Although disrupted glucose metabolism in the mPFC was detected in our present study, the level of ATP was not measured. Animals of different strains, ages, and genders may also be considered in subsequent studies. Depp1 may be associated with synaptic damage in depression along with hyperglycemia, but its specific neuronal populations and function need to be further examined. In addition, if these results could be validated in clinical trials, they could serve as a basis for precision treatment for patients with comorbidity of depression and diabetes.

## 5. Conclusions

In conclusion, our research reveals that an upregulation of Depp1 in the mPFC is closely involved in the co-occurrence of depression and diabetes. Further identification of the biomarkers that significantly correlated with the expression level of DEPP1 in the mPFC will benefit the diagnosis of possible comorbidity in depression and diabetes and will facilitate the research and development of an agent to achieve targeting effects.

## Data Availability

Data are contained within the article or Supplementary Materials.

## Supplementary Materials

The following supporting information can be downloaded at: https://www.mdpi.com/article/10.3390/metabo15010034/s1, Table S1: The raw data of LC-Q/TOF-MS-based metabolomics assays; Table S2: Adjusted *p*-values (FDR) in metabolomics analysis; Table S3: All the specific information of statistical analysis in the manuscript; Table S4: The raw data and adjusted *p*-values (FDR) in transcriptomics analysis.

## References

[1] Smith K., De Torres I. (2014). Mental health: A world of depression. Nature.

[2] Dadi A.F., Miller E.R., Bisetegn T.A., Mwanri L. (2020). Global burden of antenatal depression and its association with adverse birth outcomes: An umbrella review. BMC Public Health.

[3] Semenkovich K., Brown M.E., Svrakic D.M., Lustman P.J. (2015). Depression in type 2 diabetes mellitus: Prevalence, impact, and treatment. Drugs.

[4] Oladeji B.D., Gureje O. (2013). The comorbidity between depression and diabetes. Curr. Psychiatry Rep..

[5] Sun H., Saeedi P., Karuranga S., Pinkepank M., Ogurtsova K., Duncan B.B., Stein C., Basit A., Chan J.C.N., Mbanya J.C. (2022). IDF Diabetes Atlas: Global, regional and country-level diabetes prevalence estimates for 2021 and projections for 2045. Diabetes Res. Clin. Pract..

[6] Kimbrell T.A., Ketter T.A., George M.S., Little J.T., Benson B.E., Willis M.W., Herscovitch P., Post R.M. (2002). Regional cerebral glucose utilization in patients with a range of severities of unipolar depression. Biol. Psychiatry.

[7] Winokur A., Maislin G., Phillips J.L., Amsterdam J.D. (1988). Insulin resistance after oral glucose tolerance testing in patients with major depression. Am. J. Psychiatry.

[8] Wu G.R., Baeken C. (2022). Brainstem glucose metabolism predicts reward dependence scores in treatment-resistant major depression. Psychol. Med..

[9] Chuang J.C., Cui H., Mason B.L., Mahgoub M., Bookout A.L., Yu H.G., Perello M., Elmquist J.K., Repa J.J., Zigman J.M. (2010). Chronic social defeat stress disrupts regulation of lipid synthesis. J. Lipid Res..

[10] Li X., Qiu W., Li N., Da X., Ma Q., Hou Y., Wang T., Song M., Chen J. (2020). Susceptibility to Hyperglycemia in Rats with Stress-Induced Depressive-Like Behavior: Involvement of IL-6 Mediated Glucose Homeostasis Signaling. Front. Psychiatry.

[11] Ouyang X., Wang Z., Luo M., Wang M., Liu X., Chen J., Feng J., Jia J., Wang X. (2021). Ketamine ameliorates depressive-like behaviors in mice through increasing glucose uptake regulated by the ERK/GLUT3 signaling pathway. Sci. Rep..

[12] Patel S.S., Mehta V., Changotra H., Udayabanu M. (2016). Depression mediates impaired glucose tolerance and cognitive dysfunction: A neuromodulatory role of rosiglitazone. Horm. Behav..

[13] van der Kooij M.A., Rojas-Charry L., Givehchi M., Wolf C., Bueno D., Arndt S., Tenzer S., Mattioni L., Treccani G., Hasch A. (2022). Chronic social stress disrupts the intracellular redistribution of brain hexokinase 3 induced by shifts in peripheral glucose levels. J. Mol. Med..

[14] van der Kooij M.A., Jene T., Treccani G., Miederer I., Hasch A., Voelxen N., Walenta S., Müller M.B. (2018). Chronic social stress-induced hyperglycemia in mice couples individual stress susceptibility to impaired spatial memory. Proc. Natl. Acad. Sci. USA.

[15] Roden M., Shulman G.I. (2019). The integrative biology of type 2 diabetes. Nature.

[16] Mommersteeg P.M., Herr R., Pouwer F., Holt R.I., Loerbroks A. (2013). The association between diabetes and an episode of depressive symptoms in the 2002 World Health Survey: An analysis of 231,797 individuals from 47 countries. Diabet. Med..

[17] Kuroda Y., Kuriyama H., Kihara S., Kishida K., Maeda N., Hibuse T., Nishizawa H., Matsuda M., Funahashi T., Shimomura I. (2010). Insulin-mediated regulation of decidual protein induced by progesterone (DEPP) in adipose tissue and liver. Horm. Metab. Res..

[18] Shin D., Anderson D.J. (2005). Isolation of arterial-specific genes by subtractive hybridization reveals molecular heterogeneity among arterial endothelial cells. Dev. Dyn..

[19] Watanabe H., Nonoguchi K., Sakurai T., Masuda T., Itoh K., Fujita J. (2005). A novel protein Depp, which is induced by progesterone in human endometrial stromal cells activates Elk-1 transcription factor. Mol. Hum. Reprod..

[20] Chen S., Gai J., Wang Y., Li H. (2011). FoxO regulates expression of decidual protein induced by progesterone (DEPP) in human endothelial cells. FEBS Lett..

[21] Ragel B.T., Couldwell W.T., Gillespie D.L., Jensen R.L. (2007). Identification of hypoxia-induced genes in a malignant glioma cell line (U-251) by cDNA microarray analysis. Neurosurg. Rev..

[22] Salcher S., Hermann M., Kiechl-Kohlendorfer U., Ausserlechner M.J., Obexer P. (2017). C10ORF10/DEPP-mediated ROS accumulation is a critical modulator of FOXO3-induced autophagy. Mol. Cancer.

[23] Zhu Y.-J., Huang J., Chen R., Zhang Y., He X., Duan W.-X., Zou Y.-L., Sun M.-M., Sun H.-L., Cheng S.-M. (2024). Autophagy dysfunction contributes to NLRP1 inflammasome-linked depressive-like behaviors in mice. J. Neuroinflamm..

[24] Zhang K., Wang F., Zhai M., He M., Hu Y., Feng L., Li Y., Yang J., Wu C. (2023). Hyperactive neuronal autophagy depletes BDNF and impairs adult hippocampal neurogenesis in a corticosterone-induced mouse model of depression. Theranostics.

[25] Li W., Ji M., Lin Y., Miao Y., Chen S., Li H. (2018). DEPP/DEPP1/C10ORF10 regulates hepatic glucose and fat metabolism partly via ROS-induced FGF21. FASEB J..

[26] Wyant G.A., Jiang Q., Singh M., Qayyum S., Levrero C., Maron B.A., Kaelin W.G. (2024). Induction of DEPP1 by HIF Mediates Multiple Hallmarks of Ischemic Cardiomyopathy. Circulation.

[27] Du Y., Yan T., Wu B., He B., Jia Y. (2024). Research on the mechanism of antidepressive effect of Suanzaoren Decoction through TLR4/MyD88/NF-κB pathway and Wnt/β-catenin pathway. J. Ethnopharmacol..

[28] Freeman R. (2009). Not all neuropathy in diabetes is of diabetic etiology: Differential diagnosis of diabetic neuropathy. Curr. Diabetes Rep..

[29] Wang J., Gong B., Zhao W., Tang C., Varghese M., Nguyen T., Bi W., Bilski A., Begum S., Vempati P. (2014). Epigenetic mechanisms linking diabetes and synaptic impairments. Diabetes.

[30] Xiang Q., Tao J.S., Dong S., Liu X.L., Yang L., Liu L.N., Deng J., Li X.H. (2024). Heterogeneity and synaptic plasticity analysis of hippocampus based on db-/-mice induced diabetic encephalopathy. Psychoneuroendocrinology.

[31] Jiang T., Zhang W., Wang Y., Zhang T., Wang H., Yang Z. (2022). Rapamycin Pretreatment Attenuates High Glucose-induced Alteration of Synaptic Transmission in Hippocampal Dentate Gyrus Neurons. Neuroscience.

[32] Golden S.A., Covington H.E., Berton O., Russo S.J. (2011). A standardized protocol for repeated social defeat stress in mice. Nat. Protoc..

[33] Chiba S., Numakawa T., Ninomiya M., Richards M.C., Wakabayashi C., Kunugi H. (2012). Chronic restraint stress causes anxiety- and depression-like behaviors, downregulates glucocorticoid receptor expression, and attenuates glutamate release induced by brain-derived neurotrophic factor in the prefrontal cortex. Prog. Neuro-Psychopharmacol. Biol. Psychiatry.

[34] Herselman M.F., Lin L., Luo S., Yamanaka A., Zhou X.F., Bobrovskaya L. (2023). Sex-Dependent Effects of Chronic Restraint Stress on Mood-Related Behaviours and Neurochemistry in Mice. Int. J. Mol. Sci..

[35] Logan R.W., Edgar N., Gillman A.G., Hoffman D., Zhu X., McClung C.A. (2015). Chronic Stress Induces Brain Region-Specific Alterations of Molecular Rhythms that Correlate with Depression-like Behavior in Mice. Biol. Psychiatry.

[36] Han Y.C., Tang S.Q., Liu Y.T., Li A.M., Zhan M., Yang M., Song N., Zhang W., Wu X.Q., Peng C.H. (2021). AMPK agonist alleviate renal tubulointerstitial fibrosis via activating mitophagy in high fat and streptozotocin induced diabetic mice. Cell Death Dis..

[37] Xu J., Zheng B., Ma Y., Zhang X., Cheng J., Yang J., Li P., Zhang J., Jing L., Xu F. (2023). PI3K-AKT-mTOR signaling pathway regulates autophagy of hippocampal neurons in diabetic rats with chronic unpredictable mild stress. Behav. Brain Res..

[38] Wang H., Tan Y.Z., Mu R.H., Tang S.S., Liu X., Xing S.Y., Long Y., Yuan D.H., Hong H. (2021). Takeda G Protein-Coupled Receptor 5 Modulates Depression-like Behaviors via Hippocampal CA3 Pyramidal Neurons Afferent to Dorsolateral Septum. Biol. Psychiatry.

[39] Zhang Y., Lu W., Wang Z., Zhang R., Xie Y., Guo S., Jiao L., Hong Y., Di Z., Wang G. (2020). Reduced Neuronal cAMP in the Nucleus Accumbens Damages Blood-Brain Barrier Integrity and Promotes Stress Vulnerability. Biol. Psychiatry.

[40] Xu A., Lam M.C., Chan K.W., Wang Y., Zhang J., Hoo R.L., Xu J.Y., Chen B., Chow W.S., Tso A.W. (2005). Angiopoietin-like protein 4 decreases blood glucose and improves glucose tolerance but induces hyperlipidemia and hepatic steatosis in mice. Proc. Natl. Acad. Sci. USA.

[41] Qin Z., Shi D.D., Li W., Cheng D., Zhang Y.D., Zhang S., Tsoi B., Zhao J., Wang Z., Zhang Z.J. (2023). Berberine ameliorates depression-like behaviors in mice via inhibiting NLRP3 inflammasome-mediated neuroinflammation and preventing neuroplasticity disruption. J. Neuroinflamm..

[42] Zhuang L., Gao W., Chen Y., Fang W., Lo H., Dai X., Zhang J., Chen W., Ye Q., Chen X. (2024). LHPP in Glutamatergic Neurons of the Ventral Hippocampus Mediates Depression-like Behavior by Dephosphorylating CaMKIIα and ERK. Biol. Psychiatry.

[43] Cao X., Li L.P., Wang Q., Wu Q., Hu H.H., Zhang M., Fang Y.Y., Zhang J., Li S.J., Xiong W.C. (2013). Astrocyte-derived ATP modulates depressive-like behaviors. Nat. Med..

[44] Idle J.R., Gonzalez F.J. (2007). Metabolomics. Cell Metab..

[45] Johnson C.H., Ivanisevic J., Siuzdak G. (2016). Metabolomics: Beyond biomarkers and towards mechanisms. Nat. Rev. Mol. Cell Biol..

[46] Detka J., Kurek A., Basta-Kaim A., Kubera M., Lasoń W., Budziszewska B. (2014). Elevated brain glucose and glycogen concentrations in an animal model of depression. Neuroendocrinology.

[47] Marano C.M., Workman C.I., Lyman C.H., Kramer E., Hermann C.R., Ma Y., Dhawan V., Chaly T., Eidelberg D., Smith G.S. (2014). The relationship between fasting serum glucose and cerebral glucose metabolism in late-life depression and normal aging. Psychiatry Res..

[48] Fan J., Guo F., Mo R., Chen L.Y., Mo J.W., Lu C.L., Ren J., Zhong Q.L., Kuang X.J., Wen Y.L. (2023). O-GlcNAc transferase in astrocytes modulates depression-related stress susceptibility through glutamatergic synaptic transmission. J. Clin. Investig..

[49] Minhas P.S., Jones J.R., Latif-Hernandez A., Sugiura Y., Durairaj A.S., Wang Q., Mhatre S.D., Uenaka T., Crapser J., Conley T. (2024). Restoring hippocampal glucose metabolism rescues cognition across Alzheimer’s disease pathologies. Science.

[50] Lin S., Huang L., Luo Z.C., Li X., Jin S.Y., Du Z.J., Wu D.Y., Xiong W.C., Huang L., Luo Z.Y. (2022). The ATP Level in the Medial Prefrontal Cortex Regulates Depressive-like Behavior via the Medial Prefrontal Cortex-Lateral Habenula Pathway. Biol. Psychiatry.

[51] Lim B.K., Huang K.W., Grueter B.A., Rothwell P.E., Malenka R.C. (2012). Anhedonia requires MC4R-mediated synaptic adaptations in nucleus accumbens. Nature.

[52] Li S., Yang D., Zhou X., Chen L., Liu L., Lin R., Li X., Liu Y., Qiu H., Cao H. (2024). Neurological and metabolic related pathophysiologies and treatment of comorbid diabetes with depression. CNS Neurosci. Ther..

